# USP15 facilitates the progression of bladder cancer by amplifying the activation of the NF-κB signaling pathway

**DOI:** 10.18632/aging.205696

**Published:** 2024-03-28

**Authors:** Yun Li, Chenghang Jiang, Quanqi Liu, Pengfei Zhou, Daxue Tian, Ying Zeng, Mingfeng Xiang

**Affiliations:** 1 Department of Ophthalmology, Second Affiliated Hospital of Nanchang University, Nanchang, China; 2 Department of Emergency Medicine, Emergency and Critical Care Center, Zhejiang Provincial People’s Hospital, Affiliated People’s Hospital, Hangzhou Medical College, Hangzhou, Zhejiang, China; 3 Department of Urology, Jinhua Hospital Affiliated to Zhejiang University School of Medicine, Jinhua, China; 4 Department of Ophthalmology, The First Affiliated Hospital, Sun-Yat-sen University, Guangzhou, China; 5 Department of Urology, Second Affiliated Hospital of Nanchang University, Nanchang, China

**Keywords:** USP15, BRCC3, NF-κB signaling pathway, bladder cancer, proliferation, migration and invasion

## Abstract

USP15, a pivotal member of the deubiquitinase family, plays a crucial role in orchestrating numerous vital biological processes, including the regulation of NF-κB signaling pathway and deubiquitination of proto-oncogenes. In various cancers, USP15 has been validated to exhibit up-regulated expression, impacting the initiation and progression of cancer. However, its precise mechanism in bladder cancer remains elusive. Our study shed light on the significant overexpression of USP15 in bladder cancer cells compared to normal bladder cells, correlating with a poorer prognosis for bladder cancer patients. Strikingly, attenuation of USP15 expression greatly attenuated the proliferation, migration, and invasion of bladder cancer cells. Moreover, upregulation of USP15 was found to drive cancer progression through the activation of the NF-κB signaling pathway. Notably, USP15 directly deubiquitinates BRCC3, heightening its expression level, and subsequent overexpression of BRCC3 counteracted the antitumoral efficacy of USP15 downregulation. Overall, our findings elucidated the carcinogenic effects of USP15 in bladder cancer, primarily mediated by the excessive activation of the NF-κB signaling pathway, thereby promoting tumor development. These results underscore the potential of USP15 as a promising therapeutic target for bladder cancer in the future.

## INTRODUCTION

Bladder cancer stands as a prevalent malignancy within the urinary system, asserting its escalating morbidity and mortality rates in China with each passing year [1]. Disturbingly, the incidence of bladder cancer among younger individuals is steadily rising, with a notable predominance of male patients [2, 3]. Notably, bladder cancer represents one of the most frequently encountered urinary system tumor affecting men in the world [4]. Furthermore, advanced bladder cancer, characterized by its highly malignant nature, poses a substantial challenge due to its heightened recurrence and mortality rates [5, 6]. Consequently, the early detection, accurate prognosis, and assessment of treatment effectiveness assume paramount importance in combatting this formidable disease.

Ubiquitin-specific protease USP15, a vital constituent of the ubiquitinase family, demonstrates wide-ranging expression throughout diverse tissues and organs, with notable prominence in the testis, pancreas, bladder, and adrenal gland [7, 8]. Operating as a significant member within the USP family, USP15 exerts its crucial role by deubiquitinating proto-oncogene proteins, such as E3 ubiquitin ligase, thereby conferring stability [9, 10]. Additionally, it plays a pivotal role in regulating pivotal signaling pathways, including transforming growth factor β, p53, and nuclear factor κB [11, 12]. Impressively, USP15 has garnered attention in its association with the occurrence and progression of various malignancies, including ovarian cancer, breast cancer, and colorectal cancer [13–15]. However, scarce reports exist pertaining to the potential mechanism by which USP15 may exert its effect in bladder cancer.

NF-κB, a group of nucleoprotein genes, serves as a pivotal mediator for the expression of a wide array of genes associated with diverse biological processes including immune response, inflammation, and cell proliferation [16–18]. Comprising five distinct members, namely, RelA (also known as p65), RelB, c-RelA, NF-κB1/p50, and NF-κB2/p52, this family orchestrates the functioning of the NFκB signaling pathway, which encompasses both canonical and non-canonical pathways [19]. In the canonical pathway, phosphorylation of IκBα at two N-terminal serines by IκB kinase (IKK) initiates its ubiquitination and subsequent proteasomal degradation. This event triggers the translocation of NF-κB complexes, predominantly p50/RelA (p65) and p50/c-Rel dimers, into the nucleus [20]. Conversely, the non-classical NF-κB pathway involves distinct signaling molecules, leading to the activation of the p52/RelB dimer [21]. Recent studies have shed light on the association between BRCC3 and the activation of NF-κB signaling in bladder cancer. BRCC3, also known as the human homolog of BRCC36, serves as a deubiquitinating enzyme belonging to the JAMM/MPN+ subclass [22]. Distinguished by its possession of two histidine residues and one aspartic acid residue, BRCC3 markedly exhibits the specific ability to recognize and hydrolyze polyubiquitin chains formed by K63 site linkage [23]. Functionally, BRCC3 assumes a crucial role in diverse processes such as angiogenesis, tumorigenesis, and inflammatory response [24–26]. Regrettably, current literature lacks comprehensive reports delineating the upstream mechanism through which BRCC3 regulates NF-κB activity, warranting further investigations in this area.

In our research, we identified that USP15 was aberrantly elevated in bladder tumor tissues and associated with poorer prognosis. The expression of USP15 was associated with the proliferation, migration and invasiveness of bladder cancer. USP15 induced bladder cancer progression through reducing the degradation of BRCC3, thereby regulating the classical NF-κB signaling pathway. The *in vivo* experiments were consistent with the *in vitro* assays. Our research shows that USP15 plays an important role in bladder cancer, which may be a treated target for the disease.

## MATERIALS AND METHODS

### Patients and tumor specimens

During the period from June 2018 to August 2022, bladder tumor tissues and their corresponding adjacent tissues were meticulously gathered from willing patients who had undergone bladder tumor resection. To ensure optimal preservation of sample integrity, all collected specimens were promptly subjected to storage at −80°C. It is important to note that the Ethics Committee of the esteemed Second Affiliated Hospital of Nanchang University granted their formal approval for the execution of this study, underscoring its ethical compliance and adherence to established guidelines.

### Cell lines and cell culture

Our experiment utilized a selection of bladder cancer cell lines, specifically SV-HUC-1, 5637, T24, J82, and UM-UC-3, which were procured from the Shanghai Institute of Cell Biology, China. These cell lines were maintained at a temperature of 37°C in Petri dishes, nourished by a medium supplemented with 10% fetal bovine serum.

### TIMER database analysis

TIMER is a comprehensive resource for analysis of the level of immune aggression in various cancers (https://cistrome.shinyapps.io/timer/). In the study, “Diff-Exp module” were used to determine the expression of USP15 in different cancer type.

### Real-time quantitative PCR (qRT-PCR)

To USP15 mRNA, we employed the widely accepted Trizol-based protocol, a standardized method known for its effectiveness. Following the manufacturer’s instructions, we conducted a semi-quantitative analysis of the resulting amplification product subsequent to the USP15 amplification process. This analysis was skillfully executed using the PrimeScript RT Kit (Invitrogen, USA) in conjunction with the SYBR Premix Ex Taq (TaKaRa, Beijing, China) to ensure accurate and reliable measurements.

### Western blotting

For protein extraction, we specifically opted for cells in the logarithmic growth phase, ensuring optimal protein yield. The method used to extract the protein aligned with the text cited [27], adhering to established protocols. To determine the concentration of the extracted protein, we employed the BCA protein assay kit, a widely utilized and reputable technique. Following the addition of the appropriate buffer, the sample protein was subjected to a 15-minute boiling period. Subsequently, the protein was separated through SDS-PAGE electrophoresis, and efficiently transferred to a PVDF membrane in a timely manner. To minimize nonspecific binding, the membrane was then blocked with skimmed milk powder. The primary antibodies, including the anti-USP15 polyclonal antibody (ab71713, Abcam, UK), anti-p-P65 monoclonal antibody (ab32536, Abcam), anti-BRCC3 monoclonal antibody (ab115172, Abcam), anti-Tubulin monoclonal antibody (11224-1-AP, Proteintech, China), and anti-Ub monoclonal antibody (code sc8017, 1:500, Santa Cruz, USA), were individually added and allowed to incubate overnight in a refrigerator at 4°C. On the subsequent day, after thorough washing of the membrane, the secondary antibody was introduced. Following this, the exposure results were meticulously observed upon the addition of the luminescence reagent.

### UALCAN database analysis

The UALCAN database (http://ualcan.path.uab.edu/) is an online interactive web resource that provides available cancer transcriptome data and patient information (excerpted from TCGA). In our study, we evaluate USP15 expression in the modules “bladder cancer” dataset and “expression analysis” via UALCAN database. There was also analysis of USP15 expression in noncancerous tissues and bladder cancer samples according to clinicopathological features, such as tumor size, lymph node invasion and metastatic status.

### Constructs and plasmids

To achieve shRNA-mediated silencing of USP15, we acquired the RNA duplex, USP15, and BRCC3 plasmids from the Gene Pharma (Shanghai, China). The corresponding gene-expression vectors were obtained by skillfully inserting the cDNAs of USP15 and BRCC3 into the pCMV-Flag vector, ensuring compatibility and efficient expression. For transfection purposes, both the USP15 shRNA and shRNA were procured from Gene Pharma (Shanghai, China). Following the guidelines provided by the manufacturer (reference 26), we conducted the transfection process in bladder cancer cells using the Lipofectamine 2000 transfection reagent (Invitrogen, USA). This widely utilized reagent allowed for the efficient delivery of the shRNA and overexpression vectors into the target cells.

### Cell colony formation assays

Cell suspensions were prepared by handpicking cells in the logarithmic growth phase, ensuring their vitality and optimal conditions. These selected cells were then evenly seeded into 6-well plates, creating a conducive environment for their growth and proliferation. The supernatants were gently discarded, followed by a thorough washing process. To accurately assess cellular characteristics, the number of cell clones was diligently observed and meticulously recorded under the microscope. Furthermore, to capture a detailed and permanent record, a fixed staining technique was applied, further enhancing the visibility and clarity of the cell clones.

### *In vitro* migration and invasion assays

For the migration and invasion assays, we employed the highly reliable and widely used Transwell chambers. In the migration assay, we introduced 6 × 10^4^ cells into the upper chamber, ensuring the removal of animal serum to accurately assess cell migration. Conversely, in the invasion assay, the upper chamber was precoated with Matrigel, and 1 × 10^5^ cells were seeded to evaluate their invasive potential. After 24 and 48 hours of monitored culture, the cells in the lower chamber were fixed and subjected to staining procedures, specifically tailored for migration and invasion assays, respectively. To capture the results, we employed a microscope DP70 CCD system (Olympus Corp., Tokyo, Japan), capturing random snapshots of cells in 15 fields of view. This approach allowed for a representative sampling and provided a visually compelling representation of the experimental outcomes.

### LinkedOmics analysis

LinkedOmics is a publicly available portal consisting of 32 TCGA cancer-related cubes.

By analyzing genome-wide expression profiles, GSEA can identify disease-related genes or proteins that range from a large number of genes or proteins. GSEA is different from the traditional enrichment analysis, which is not restricted to the differential genes, so sometimes the analysis results are more comprehensive and reliable. It can be used to find distinct gene-enriched pathways in a predetermined gene set. KEGG is a huge pathway database with powerful graphic functions, which can enable a systematic analysis of gene product function and its metabolic pathways in the cell.

### Animal experiments

The mice were ethically divided into two distinct groups, ensuring a well-controlled experimental setup. In each group, an equal number of J82 cells transfected with either shNC or shUSP15 were carefully injected. To monitor the tumor growth, the size of the tumors was methodically measured and accurately recorded on a weekly basis. This was achieved using a vernier caliper, and the tumor volume was calculated using the formula V = (length/2) × (width^2^). At the conclusion of the experiment, the tumors from all mice were excised to allow for further comparison. The difference in tumor weight between the two groups of mice was then precisely assessed, shedding light on the impact of shUSP15 on tumor growth. Importantly, all animal experimentation procedures strictly adhered to the ethical standards and were approved by the Animal Experiment Ethics Committee of the Second Affiliated Hospital of Nanchang University, ensuring the welfare and ethical treatment of the laboratory animals.

### Data analysis

All data analysis in this article was conducted using the SPSS 22.0 software, a widely accepted statistical analysis tool. For normally distributed data, we employed ANOVA to evaluate the differences, while for non-parametric data, we utilized appropriate non-parametric analysis methods. In accordance with statistical conventions, a *p*-value less than 0.05 was considered statistically significant, indicating a meaningful difference between the data groups.

## RESULTS

### USP15 is abnormally up-regulated in human bladder tumor tissues

**Figure 1:**
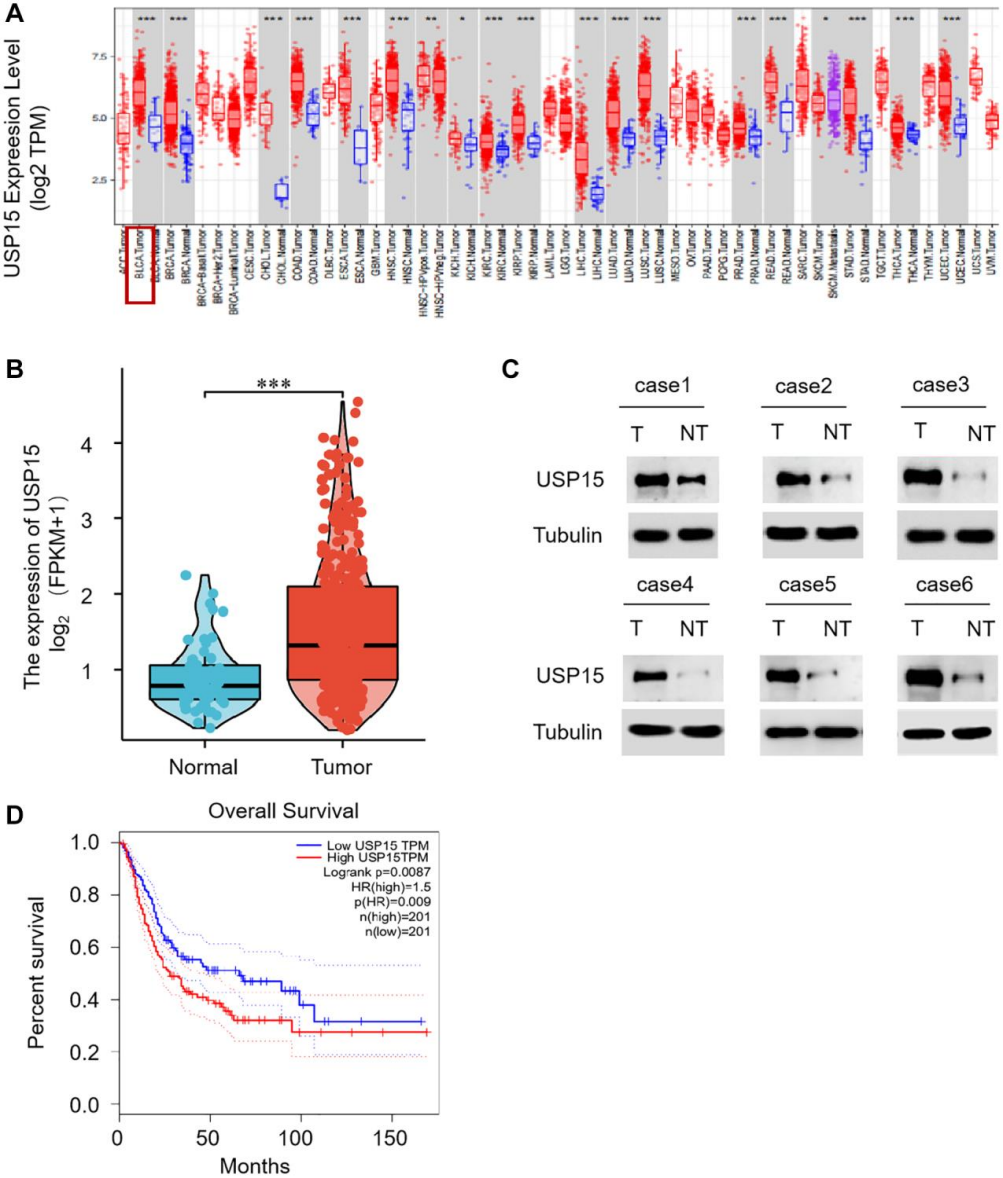
**USP15 expression is apparently upregulated in bladder cancer tissues.** (**A**) Human USP15 expression levels in different tumor types from TCGA database were determined by TIMER (^*^p < 0.05, ^**^p < 0.01, ^***^p < 0.001). (**B**) Differential USP15 mRNA expression between bladder cancer tissues and normal tissues was determined by qRT-PCR. (**C**) Determination and quantification of USP15 protein levels in BCA tissues and paired non-tumor tissues by Western blotting (*n* = 50, ^***^p < 0.001). (**D**) Analysis of the relationship between overall survival rate and gene expression, based on high (*n* = 201) or low (*n* = 201) USP15 expression in TCGA patients.

To comprehensively investigate the role of USP15 in bladder tumors, we conducted an analysis of USP15 expression in pan-cancer tissues utilizing the TIMER database. Remarkably, our results revealed a significant expression of USP15 in bladder cancer tissues (Figure 1A). Further exploration involved quantifying USP15 mRNA levels in non-tumor bladder tissues, which unveiled a comparatively higher expression of USP15 in bladder tumors (Figure 1B). To validate these findings, we performed Western blotting to assess the protein levels of USP15 in both bladder tumor tissue and adjacent normal tissue. Encouragingly, the results demonstrated an upregulation of USP15 protein expression in bladder cancer tissues compared to normal tissues (Figure 1C). Strikingly, our investigation also unveiled a noteworthy correlation between high USP15 expression and poorer overall survival outcomes (Figure 1D). Collectively, these findings provide compelling evidence that USP15 is significantly dysregulated in bladder cancer and is associated with an unfavorable prognosis.

### USP15 is associated with clinical features of bladder cancer

**Figure 2:**
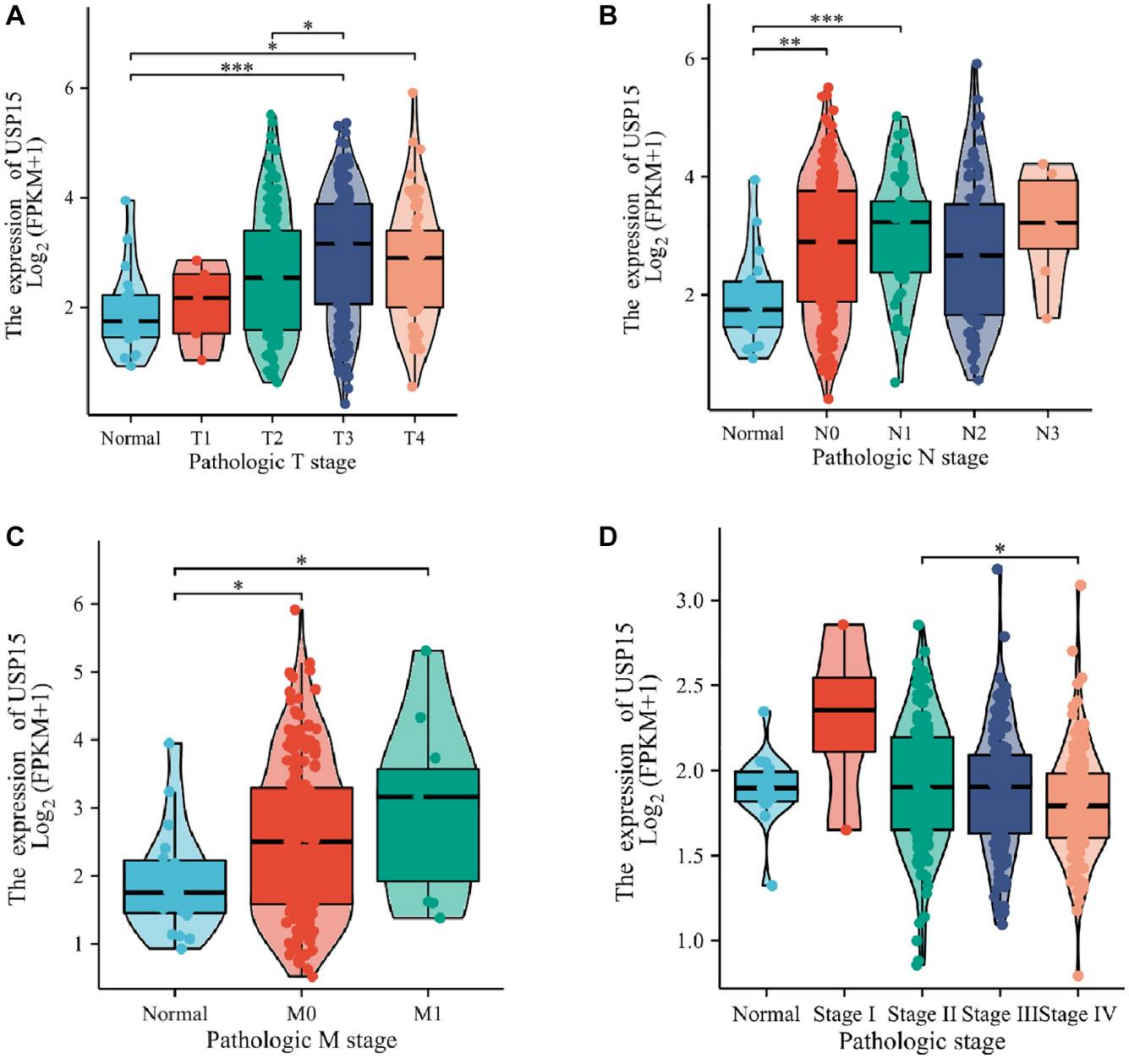
**USP15 expression is associated with clinicopathological characteristics in patients with bladder cancer.** Increased USP15 expression was significant with (**A**) tumor size. (**B**) Node. (**C**) Metastasis. (**D**) Stage.

To delve deeper into the correlation between USP15 expression and clinicopathological features in bladder tumors, we conducted an investigation to examine the association between USP15 expression and the progression of clinicopathological features. The results revealed a positive correlation between USP15 expression and TNM stage, which encompasses tumor size, lymph node involvement, and metastasis, in bladder cancer patients (Figure 2A–2C). Moreover, the expression level of USP 15 was different in the tissues of bladder cancer patients with different stages (Figure 2D).

Consequently, our findings strongly indicate that USP15 is considerably upregulated in bladder cancer tissues and exhibits a significant correlation with various clinical features of bladder cancer.

### USP15 can promote the multiplication, migration and invasion of bladder carcinoma cells *in vitro*

**Figure 3:**
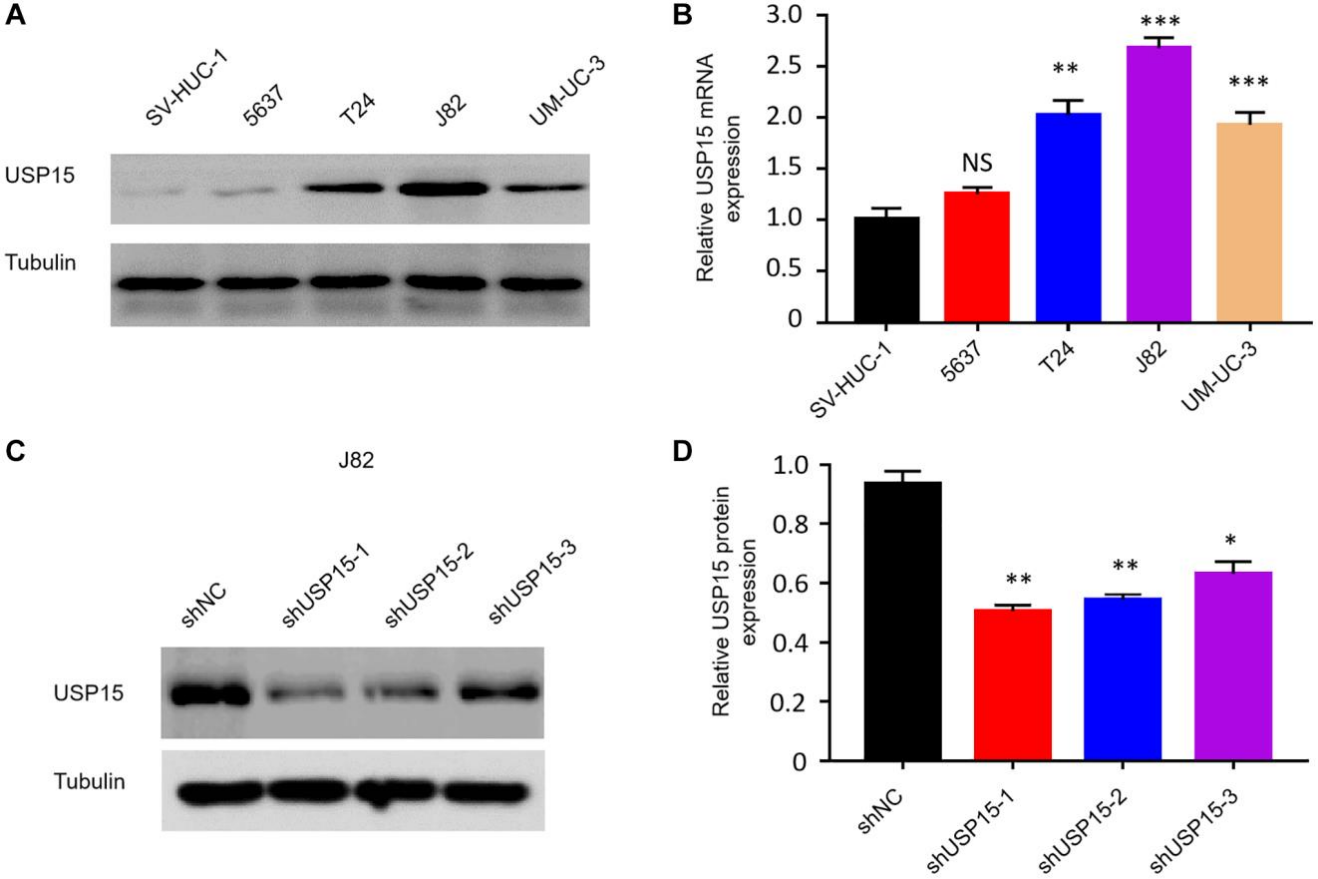
**Screening of appropriate bladder cancer cell lines and effective shRNA interference fragment of USP15.** (**A**, **B**) Western blot and qRT-PCR analysis of USP15 expression in human bladder normal cell line and bladder cancer cell lines. (**C**, **D**) USP15 protein expression in three types of shRNA.

To elucidate the correlation between USP15 expression and the proliferation of bladder tumor cells, we conducted initial investigations by examining USP15 expression levels in both normal human bladder cells (SV-HUC-1) and various bladder cancer cell lines (5637, T24, J82, and UM-UC-3) using Western blot analysis. As anticipated, the results demonstrated noticeably higher levels of USP15 protein expression in bladder cancer cells (T24, J82, and UM-UC-3) compared to normal bladder cells (Figure 3A, 3B). Subsequently, we employed effective short hairpin RNA (shRNA) interference fragments to construct recombinant plasmids for USP15 inhibition, resulting in a significant downregulation of USP15 protein expression in the USP15-inhibited group compared to the control group (Figure 3C, 3D).

**Figure 4:**
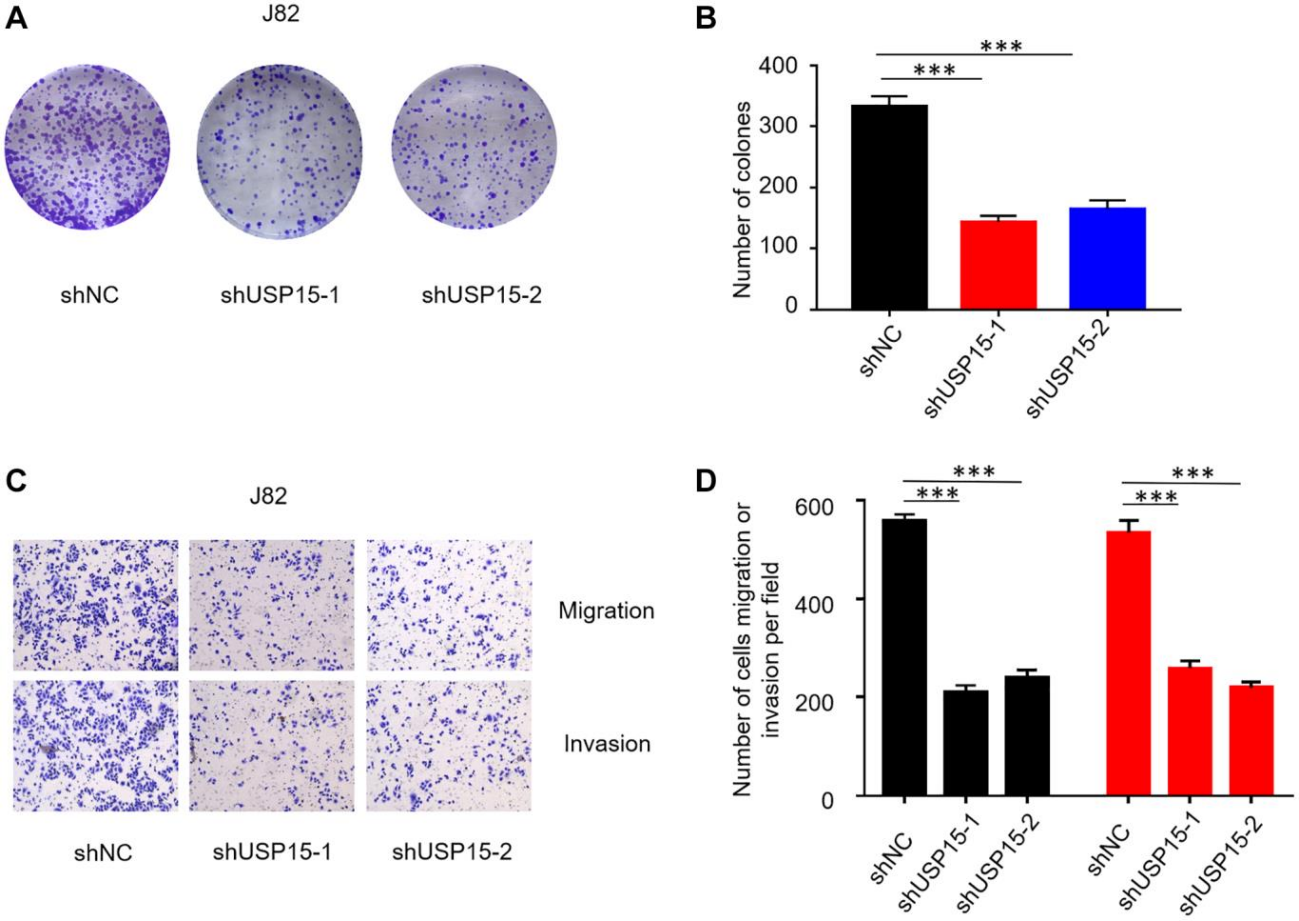
**USP15 knockdown suppresses bladder cancer cell proliferation, migration, and invasion *in vitro*.** (**A**, **B**) Proliferation capacity of bladder cancer cells J82 treated with sh-NC or sh-USP15 was detected by colony formation assays (^***^p < 0.001; scale bar, 50 μm). (**C**, **D**) Invasion and migration assays were conducted to evaluate the effect of USP15 knockdown on the metastatic ability of bladder cancer cells J82 (Magnification 200X). (^*^p < 0.05, ^**^p < 0.01, ^***^p < 0.001).

Furthermore, to investigate the impact of USP15 expression on the growth of bladder cancer cells, we individually transfected two types of shRNA into J82 cells. Subsequent colony formation assays revealed a substantial decrease in the growth ability of bladder tumor cells upon downregulation of USP15 expression, as compared to the control group (Figure 4A, 4B). Moreover, we employed Transwell migration and invasion assays to ascertain the effect of USP15 inhibition on the migratory and invasive properties of bladder tumor cells. The results demonstrated a significant inhibition of both migration and invasiveness in bladder tumor cells following USP15 inhibition (Figure 4C, 4D). In summary, our findings provide compelling evidence demonstrating the critical role of USP15 in promoting the growth, migration, and invasion of bladder tumor cells *in vitro*.

### USP15 promotes bladder cancer proliferation *in vivo*

**Figure 5:**
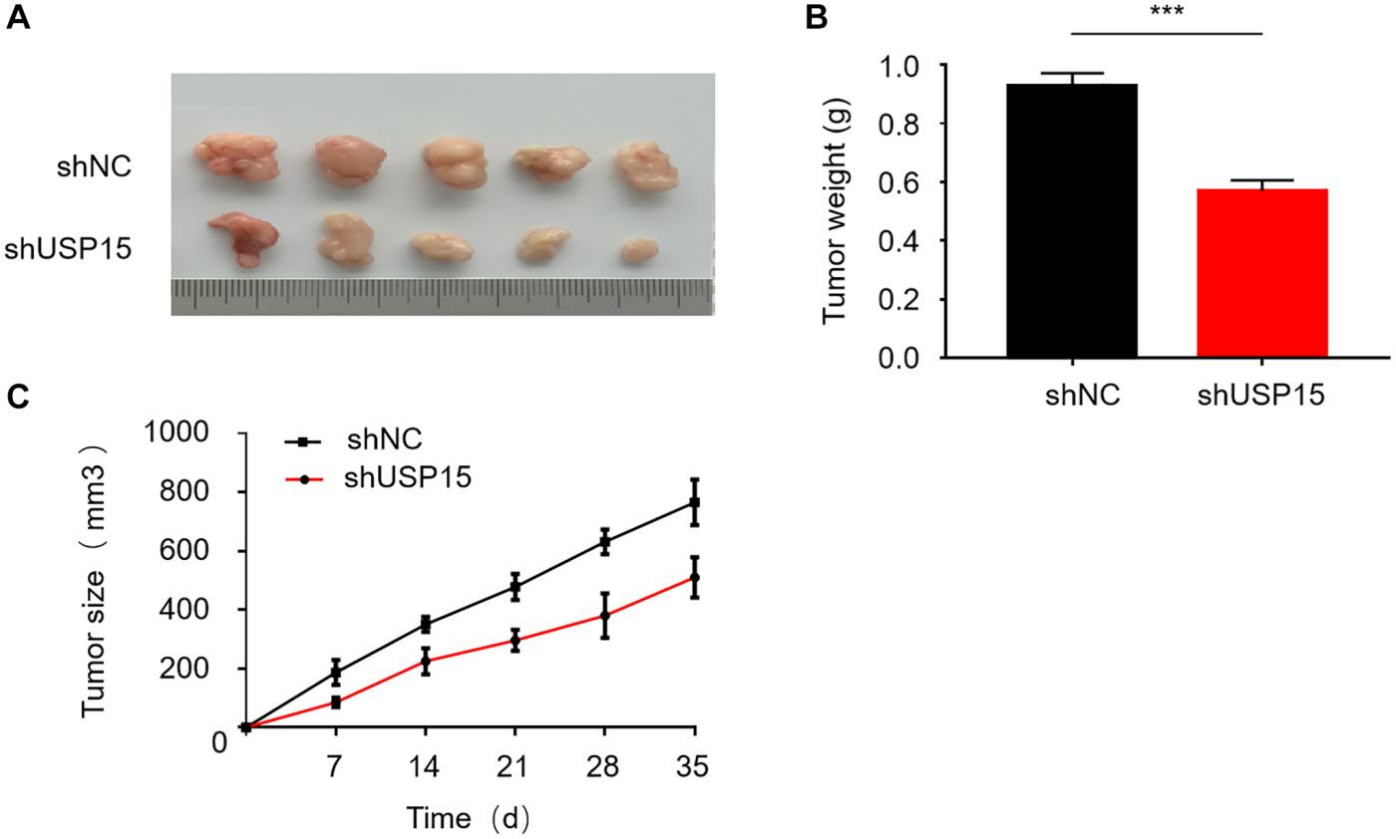
**USP15 plays an important role in bladder cancer proliferation *in vivo*.** (**A**) Measure of the tumor size of shNC or shUSP15 nude mice. (**B**) The down-regulation of USP15 significantly reduced the weight of the tumor (^***^p < 0.001). (**C**) The down-regulation of USP15 significantly reduced the tumor size (^**^p < 0.01).

To comprehensively study the impact of USP15 on bladder tumors *in vivo*, we established nude mouse models for conducting tumor experiments. The mice were divided into two randomly assigned groups, with one group receiving bladder cancer cells transfected with shUSP15 and the other group receiving bladder cancer cells transfected with shNC. Throughout the experiment, we regularly measured and compared the changes in tumor size between the two groups of mice. Remarkably, noticeable disparities were observed in both the weight and volume of tumors between the two groups. The tumors in the group that received USP15 silencing exhibited significantly reduced dimensions compared to the tumors in the control group (Figure 5A–5C). These findings elucidate that downregulating USP15 expression can effectively inhibit the growth of bladder cancer. Overall, our experimental results provide compelling evidence supporting the notion that curtailing USP15 expression holds the potential to impede the progression of bladder cancer.

### USP15 can promote the proliferation and invasiveness of bladder cancer by mediating the NF-κB signaling pathway

**Figure 6:**
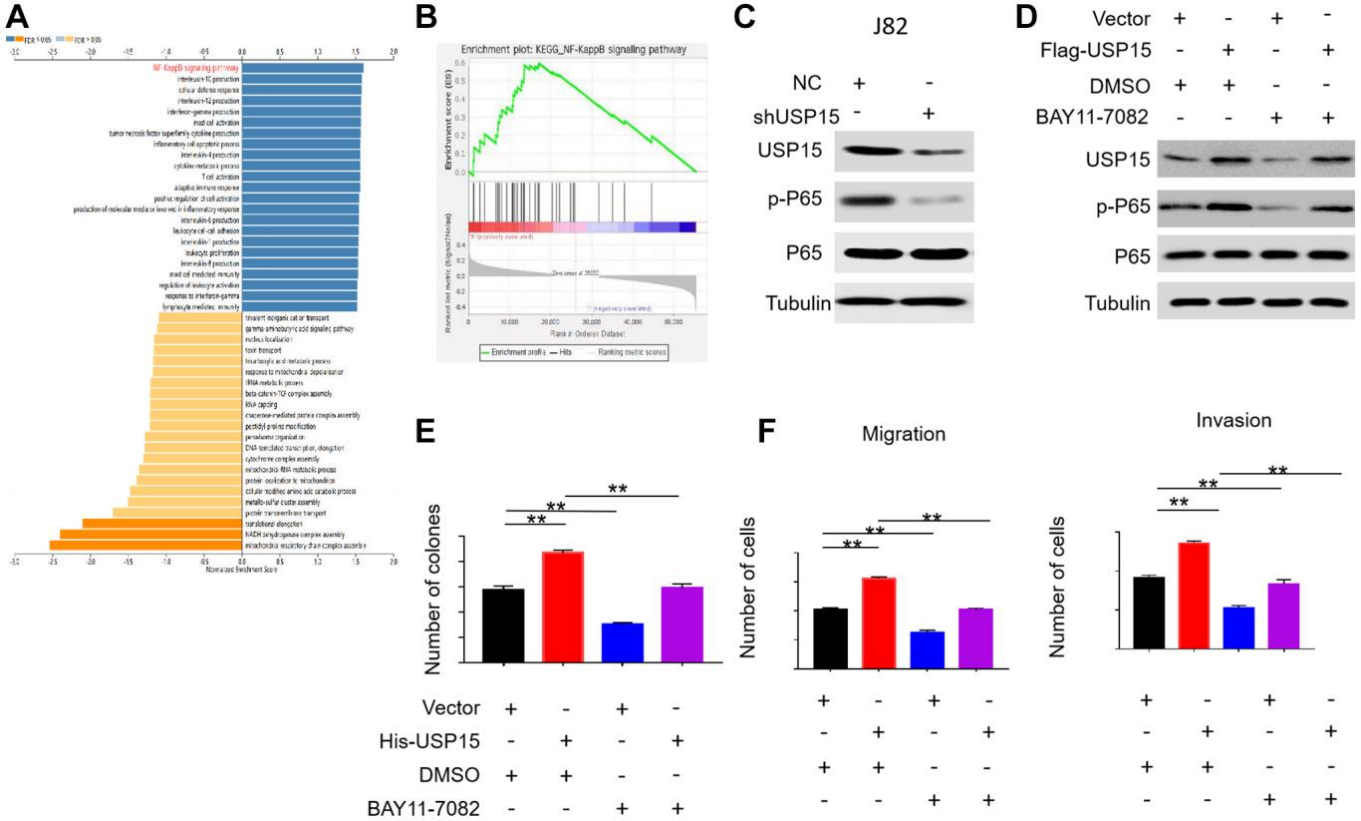
**USP15 regulates bladder cancer cell growth and invasion through NF-κB signaling pathway.** (**A**) LinkedOmics was used to analyze KEGG pathways of USP15 in bladder cancer. The NF-κB signaling pathway is marked red. FDR, false discovery rate. (**B**) GSEA results showed that the samples with high expression of USP15 had high activation of NF-κB signal pathway. (**C**) Western blotting was used to detect the expression of USP15 and p-P65 proteins in J82 cells stably transfected with control shNC RNA or USP15 shRNA. (**D**) Western blotting was used to detect the expression of USP15 and p-P65 proteins in J82 cells transfected with Flag-USP15 and with the treatment of BAY11-7082. (**E**) The proliferation of J82 cells treated with His-USP15 was detected by colony formation test (^**^p < 0.01). Treatment with BAY11-7082 significantly reduced the proliferation of J82-His-USP15 cells (^**^p < 0.01). (**F**) Treatment with BAY11-7082 significantly reduced the migration and invasion of J82-His-USP15 cells (^**^p < 0.01).

To further delve into the functional enrichment of USP15 in bladder cancer, we utilized LinkedOmics for GSEA analysis. The findings revealed a positive correlation between USP15 expression and the NF-κB pathway, which is known to play a crucial role in driving bladder cancer development (Figure 6A, 6B). This led us to hypothesize that USP15 might impact bladder tumor progression by influencing the NF-κB pathway. In subsequent experiments, we observed downregulation of USP15 expression and a subsequent reduction in p-P65 expression in J82 cells, further indicating that USP15 positively mediates the NF-κB pathway (Figure 6C). To further validate the regulatory role of USP15 in NF-κB pathway activity, we introduced the NF-κB signaling pathway inhibitor BAY11-7082 to the bladder cancer cell group with heightened USP15 expression. Interestingly, we found that the increase in p-P65 protein levels in J82 cells caused by USP15 overexpression was counteracted by the inhibitor of the NF-κB signaling pathway (Figure 6D). Additionally, our findings demonstrated that inhibition of the NF-κB pathway could partially counteract the heightened proliferation, metastasis, and invasive abilities of bladder cancer cells induced by USP15 overexpression (Figure 6E, 6F). Collectively, these results strongly suggest that USP15 promotes the proliferation, invasion, and migration of bladder cancer cells through the NF-κB pathway.

### USP15 can activate NF-κB signaling pathway via BRCC3, thereby promoting the growth, migration and invasiveness of bladder cancer

Moreover, our study aimed to elucidate the regulatory role of USP15 in the activation of the NF-κB pathway in bladder cancer. Previous studies have indicated that elevated levels of BRCC3 protein can lead to the hyperactivation of the NF-κB pathway in bladder cancer [28]. Based on this knowledge, we hypothesized that USP15 may promote NF-κB pathway activation by upregulating the expression of BRCC3.

**Figure 7:**
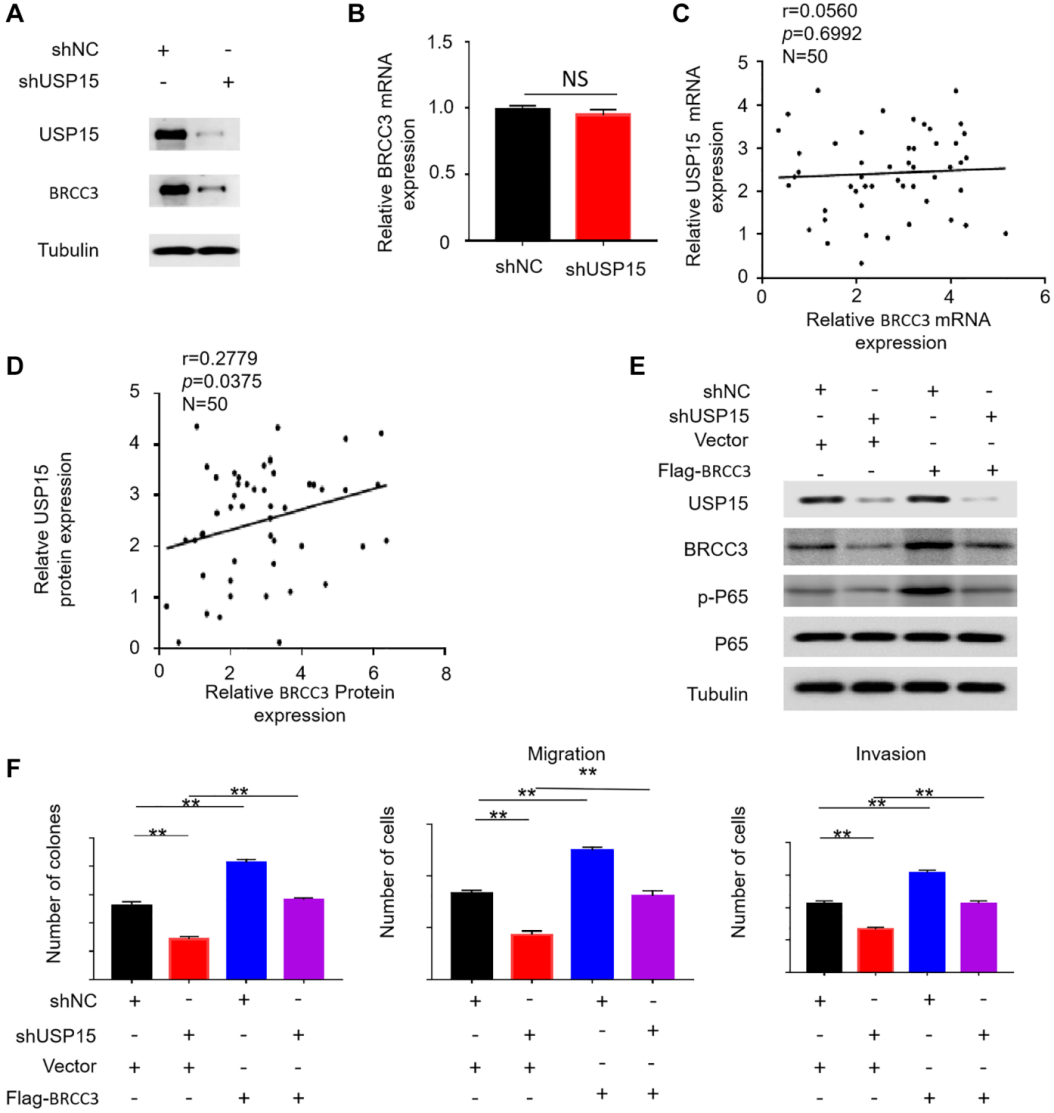
**USP15 activates NF-κB signaling pathway by upregulating BRCC3 protein expression.** (**A**) Western blotting was used to detect the expression of USP15 and BRCC3 proteins in J82 cells stably transfected with control shNC RNA or USP15 shRNA. (**B**) Relative mRNA expression of BRCC3 in normal and bladder cancer tissues. (**C**, **D**) Scatter plot of correlation between BRCC3 expression and USP15 expression. (**E**) Western blotting was used to detect the expression of USP15, BRCC3, P65 and p-P65 proteins in J82 cells stably transfected with USP15 shRNA or Flag-BRCC3 or both. (**F**) The upregulation of BRCC3 significantly saved the proliferation of J82-shUSP15 cells (^**^p < 0.01). The upregulation of BRCC3 significantly saved the migration and invasion of J82-shUSP15 cells (^**^p < 0.01).

To validate our hypothesis, we performed Western blotting to measure the amount of BRCC3 protein in bladder cancer cells with low USP15 expression. Intriguingly, we observed a corresponding downregulation of BRCC3 protein expression when USP15 expression was inhibited (Figure 7A). However, we did not observe a similar downregulation of BRCC3 mRNA expression when USP15 expression was downregulated (Figure 7B). Additionally, correlation analysis revealed a correlation between USP15 and BRCC3 at the protein expression level, but not at the mRNA level (Figure 7C, 7D).

In order to further investigate the relationship between USP15 and BRCC3, we conducted additional Western blotting experiments. We established four cell groups: a normal control group, a group with low USP15 expression and normal BRCC3 expression, a group with normal USP15 expression and high BRCC3 expression, and a group with inhibited USP15 expression and excessive BRCC3 expression. The Western blot results indicated that the expression of p-P65 in the fourth group was lower than that of cells with only BRCC3 overexpression but stronger than that of cells with only reduced USP15 expression (Figure 7E). This finding suggests that increased BRCC3 expression rescued the decrease in p-P65 expression caused by low USP15 expression. These results demonstrate that USP15 can activate the NF-κB pathway by modulating the levels of BRCC3.

To investigate whether USP15 can affect the growth, migration, and invasiveness of bladder cancer by regulating BRCC3, we generated bladder cancer cell models with different expression levels of USP15 and BRCC3 and evaluated their proliferative, migratory, and invasive capabilities. The results revealed that excessive expression of BRCC3 promoted the growth, invasion, and migration of bladder cancer cells and also counteracted the reduced proliferation and invasion caused by the inhibition of USP15 expression (Figure 7F). In summary, our study demonstrates that USP15 can activate the NF-κB signaling pathway through BRCC3, thus enhancing the proliferation, migration, and invasiveness of bladder cancer cells.

### USP15 can regulate the expression of BRCC3 by deubiquitinating it

**Figure 8:**
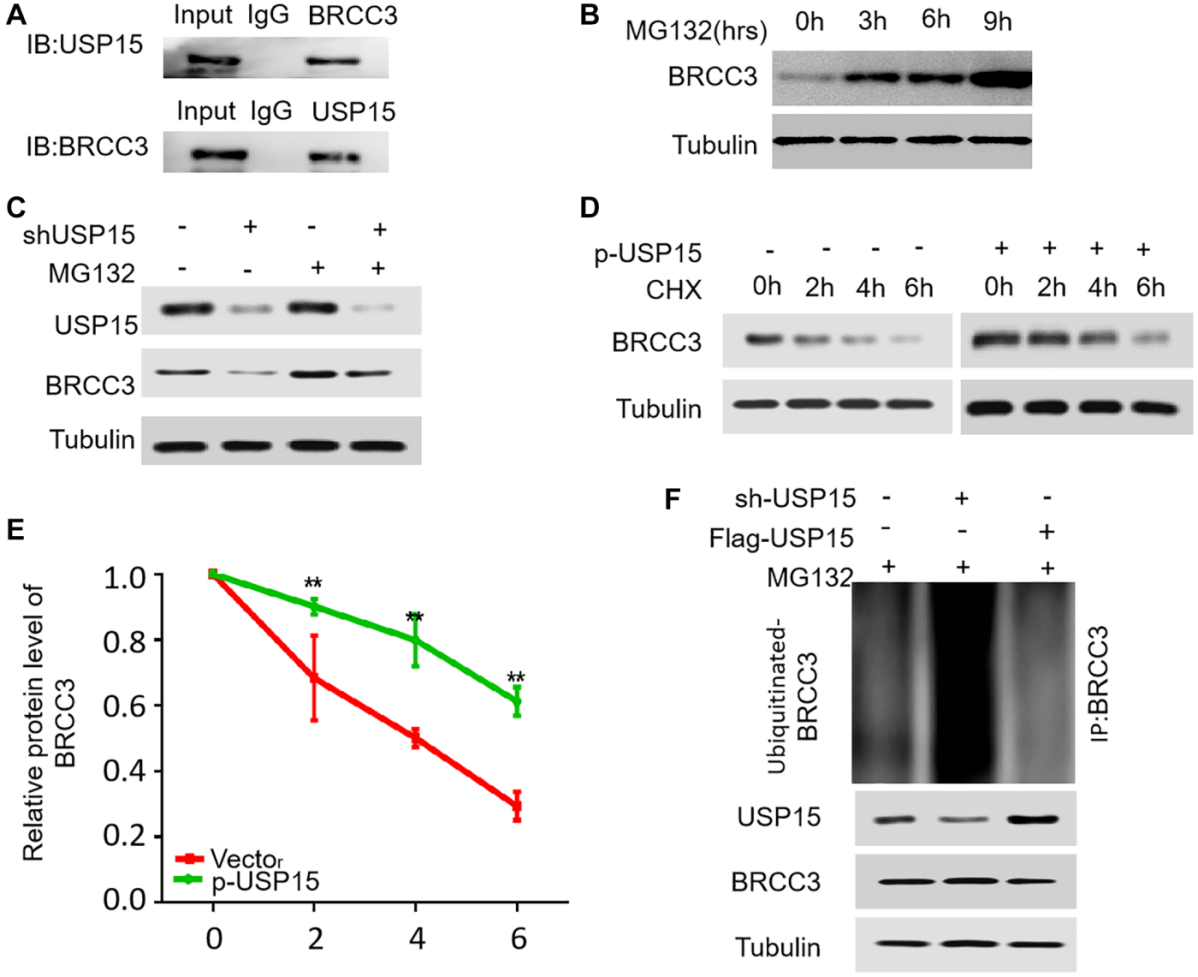
**USP15 stabilizes BRCC3 through deubiquitination.** (**A**) Co-IP between endogenous BRCC3 and USP15 in J82 cells. (**B**) J82 cells were treated with 15 μM proteasomal inhibitor MG132 for the indicated time, and the protein levels of BRCC3 were then detected. (**C**) J82 cells transfected with USP15 shRNA were treated with MG132 (15 μM). Cells were collected at 6 h and immunoblotted with the antibodies indicated. (**D**) J82 cells were transfected with or without p-USP15, and treated with cycloheximide (CHX). Cells were collected at different time points and immunoblotted with the antibodies indicated. (**E**) Quantitative results of relative BRCC3 protein levels in D (^**^p < 0.01). (**F**) The knockdown or exogenous expression of USP15 altered the ubiquitination of BRCC3 in J82 cells treated with MG132 (15 μM). The levels of ubiquitin-attached BRCC3 were detected by Western blotting analysis with Ub antibody.

To elucidate the potential regulatory role of USP15 in the degradation of BRCC3 protein, our study embarked on a series of experiments. Initially, we sought to establish the direct interaction between endogenous BRCC3 and USP15 in J82 cells through Co-IP experiments. Remarkably, our results unequivocally demonstrated their binding affinity (Figure 8A). Subsequently, we introduced the proteasome inhibitor MG132 to gauge the impact on endogenous BRCC3 protein levels in J82 cells, observing a gradual accumulation over time (Figure 8B). These intriguing findings shed light on the notion that BRCC3 is susceptible to ubiquitin-mediated degradation in bladder cancer cells. Considering these findings, we delved deeper into the quest to ascertain the influence of USP15 on BRCC3 degradation. Introducing USP15 shRNA plasmids into J82 cells, we systematically evaluated the effect of differing levels of USP15 on BRCC3. The inhibitory effect of reduced USP 15 expressions on BRCC3 expression was partially reversed under the treatment of MG132 (Figure 8C). Furthermore, by employing CHX as a translation inhibitor and monitoring BRCC3 protein content within a 0–6-hour timeframe, we uncovered a compelling correlation. Notably, overexpression of USP15 effectively decelerated the degradation rate of BRCC3, enhancing its stability, while decreased USP15 expression yielded the opposite effect (Figure 8D, 8E). Meticulously analyzing the data led to the realization that inhibition of USP15 significantly improved the ubiquitination level of BRCC3 in J82 cells, while USP15 overexpression diminished BRCC3 ubiquitination levels (Figure 8F). Thus, our results compellingly suggest that by modulating the ubiquitination of BRCC3, USP15 contributes to enhancing the stability of BRCC3 in bladder cancer cells. In conclusion, our study found a new significant mechanism that USP15 regulates the expression of NF-κB through BRCC3, resulting in increased proliferation of bladder cancer cells.

## DISCUSSION

Bladder cancer, being one of the most prevalent urinary system malignancies, presents a considerable incidence rate and a tendency for recurrence [29]. This form of cancer predominantly affects middle-aged and elderly men, often manifesting as painless hematuria [30]. Detecting bladder cancer at an early stage and promptly initiating treatment can significantly enhance the chances of survival. Hence, it remains of utmost importance to explore novel and emerging treatment modalities specifically tailored for bladder cancer.

Within cellular pathways crucial for cell proliferation, development, and innate immunity, USP15 assumes a substantial role [31, 32]. Its involvement in cancer progression, including lung cancer, has been well-documented [33, 34]. Furthermore, USP15 has shown upregulation in various other cancer types, such as glioblastoma, breast cancer, ovarian cancer and gastric cancer, to name a few [35–37]. Depending on the specific malignancy, USP15 has been reported to exert either tumor-promoting or tumor-inhibiting effects [38]. Despite a wealth of research on the oncogenic functions of USP15 in various malignancies, scant attention has been given to understanding its precise role in bladder cancer.

For the first time, our study unveils the remarkable disparity in USP15 expression between bladder tumor tissues and adjacent noncancerous tissues, with higher levels detected in the former. Importantly, this heightened USP15 expression is indicative of a shorter overall survival time. Significantly, our investigations provide unprecedented evidence of USP15’s abundant presence in both *in vivo* and *in vitro* bladder tumor tissues. Moreover, this heightened USP15 expression correlates with enhanced growth, migration, and invasion capacity of bladder tumor cells. In addition, we have shed light on one of its downstream targets and potential regulatory mechanisms. In summary, our research underscores the influential role of USP15 in shaping the progression and prognosis of bladder cancer.

Furthermore, we have delved into the potential mechanisms through which USP15 operates in bladder cancer. The NF-κB pathway, recognized as a promoter of bladder cancer development, assumes a central position in this context [39, 40]. Notably, p-P65, a key molecule involved in this carcinogenic process, emerges as a significant player [41]. Activated NF-κB, in turn, inhibits apoptosis and instigates proliferation, migration, and invasion of bladder cancer cells [42]. Our experimental findings suggest that USP15 effectively modulates the NF-κB pathway, thereby amplifying bladder cancer cell proliferation through its influence on p-P65 protein expression. To begin with, USP15 exerts a substantial impact on the activity of the NF-κB pathway in bladder cancer cells. Moreover, the downregulation of USP15 expression in these cells results in a concomitant decrease in p-P65 expression. Notably, pharmacological inhibition of the NF-κB pathway attenuates the overexpression of p-P65 in USP15-overexpressing bladder tumor cells. This inhibitory effect restrains the heightened activity of the NF-κB signaling pathway, ultimately curbing tumor proliferation and invasion in the bladder. Taken together, our comprehensive analysis establishes USP15 as a promoter of bladder cancer cell multiplication and invasion, primarily achieved through its activation of the NF-κB pathway.

Moreover, of utmost significance, our research has uncovered the regulatory mechanism by which USP15 influences the NF-κB pathway in bladder cancer—a breakthrough in our understanding of this complex interplay. Furthermore, we have made a novel discovery regarding the interrelation between USP15 and BRCC3, unearthing a previously unrecognized association. Through its regulation of BRCC3 expression, USP15 exerts control over the multiplication, invasiveness, and migration of bladder cancer cells.

It is worth noting that scientific investigations have established the up-regulation of BRCC3 protein as a catalyst for the hyperactivation of the NF-κB pathway, thereby driving bladder cancer progression [28]. Our experiments have delineated a positive correlation between USP15 and BRCC3 protein expression levels, while mRNA expression levels appear unrelated. This indicates that USP15’s regulation of BRCC3 occurs primarily at the protein level. Subsequently, through a series of comprehensive gain and loss-of-function experiments, we have reaffirmed the positive correlation between BRCC3 protein expression and USP15, confirming the pivotal role of USP15 in modulating the multiplication, invasiveness, and migration of bladder cancer by regulating BRCC3 expression.

Subsequently, to assess the interaction between USP15 and BRCC3, we conducted meticulous Co-IP experiments, conclusively demonstrating direct binding between the two proteins. However, the precise mechanistic details of this interaction remain elusive. Notably, it is highly likely that USP 15 and USP 9X, as members of the same family, have similar biological functions. Compelling research indicates that USP9X counteracts the degradation of BRCA1 via the ubiquitin-proteasome pathway [43]. BRCA1 is a crucial component of the BRCC3 complex, leading to our conjecture that USP15 may activate the NF-κB pathway by deubiquitinating BRCC3, thereby augmenting its expression and ultimately influencing bladder cancer progression. Expanding on our investigations, we conducted further research and found that up-regulation of USP15 expression decelerates the degradation rate of BRCC3. Furthermore, we observed a notable reduction in BRCC3 ubiquitination levels upon USP15 upregulation. Thus, our comprehensive findings unveil, for the first time, the association between USP15 and the ubiquitinated protease degradation of BRCC3.

In summary, we have uncovered the hitherto unexplored role of USP15 in bladder cancer, documenting its overexpression in bladder cancer tissues. Evidently, USP15 promotes the development of bladder cancer by stabilizing BRCC3, thereby activating the NF-κB signaling pathway. These groundbreaking findings suggest that USP15 holds immense promise as a potential biomarker for bladder cancer patients, while its regulation mechanism may emerge as a prospective therapeutic target for bladder cancer treatment in the future.
